# Follicular Lymphoma Presenting as Refractory Panniculitis-like Lesions: A Case Report of Successful Salvage with Autologous Stem Cell Transplantation

**DOI:** 10.3390/jcm15218369

**Published:** 2026-10-27

**Authors:** Kristina Alaiyoud, Nikolett Farmasi, Zsófia Miltényi, Judit Bedekovics, Árpád Illés, Ádám Jóna

**Affiliations:** 1 Department of Hematology, Faculty of Medicine, University of Debrecen, Nagyerdei krt. 98, 4032 Debrecen, Hungary; kristinaalaia369@gmail.com (K.A.); miltenyi.zsofia@med.unideb.hu (Z.M.); illes.arpad@med.unideb.hu (Á.I.); 2 Doctoral School of Clinical Medicine, University of Debrecen, member of ERN-EuroBloodNet (European Reference Network on Rare Haematological Diseases), 4032 Debrecen, Hungary; 3 Division of Clinical Immunology, Institute of Internal Medicine, Faculty of Medicine, University of Debrecen, 4032 Debrecen, Hungary; farmasi.nikolett@med.unideb.hu; 4 Department of Pathology, Faculty of Medicine, University of Debrecen, 4032 Debrecen, Hungary; bedekovics.judit@med.unideb.hu

**Keywords:** follicular lymphoma, mesenteric panniculitis, cutaneous involvement, extranodal disease, autologous stem cell transplantation

## Abstract

**Background:** Follicular lymphoma can be difficult to diagnose when extranodal manifestations, particularly cutaneous lesions, mimic inflammatory disease. **Case Presentation:** We report the case of a 52-year-old man with type 2 diabetes mellitus who presented with marked unintentional weight loss, iron deficiency anemia, elevated inflammatory markers, an approximately 100 mm bundle-like infiltration of the mesenteric adipose tissue, and panniculitis-like scalp lesions. Initial gastroenterological evaluation did not reveal malignancy. Biopsy of a cutaneous lesion showed a lobular lymphocytic infiltrate without evidence of malignancy, and IgG4-related mesenteric panniculitis was suspected. Despite treatment with colchicine, corticosteroids, and sequential immunosuppressive therapy, the cutaneous and subcutaneous lesions progressed. Re-evaluation revealed cervical lymphadenopathy, and histopathological examination of an excised lymph node established the diagnosis of grade 3A FL. Staging 18F-FDG PET/CT showed cervical and supraclavicular nodal disease, involvement of the nasal cavity and nasopharynx, and hypermetabolic cutaneous lesions. Six cycles of R-CHOP produced a partial metabolic response, with residual FDG-avid extranodal disease. Repeat biopsy confirmed persistent FL without evidence of large B-cell transformation. The patient subsequently received platinum- and cytarabine-based salvage chemotherapy and radiotherapy to the residual disease sites, followed by autologous stem cell transplantation after R-BEAM conditioning. On day +100 after transplantation, PET/CT showed complete metabolic remission, and the cutaneous lesions had healed. **Conclusions:** This case demonstrates that systemic FL may mimic mesenteric panniculitis and inflammatory skin disease. Persistent panniculitis-like lesions that do not respond to immunosuppressive treatment should prompt diagnostic reassessment, repeat biopsy, and careful clinicopathological correlation.

## 1. Introduction

Follicular lymphoma (FL) is one of the most common indolent B-cell non-Hodgkin lymphomas in adults. It arises from germinal center B cells and typically exhibits a follicular growth pattern and a germinal center immunophenotype, including expression of B-cell markers, CD10, and BCL6, with frequent BCL2 overexpression [1,2,3]. Current classification systems, including the fifth edition of the World Health Organization (WHO) classification and the International Consensus Classification, emphasize an integrated diagnostic approach that combines morphology, immunophenotype, genetic findings, and clinical context [4,5].

FL usually presents with painless generalized lymphadenopathy and often involves the bone marrow. Primary extranodal presentation is considerably less common [1,6]. Although systemic FL may involve the skin, cutaneous disease rarely constitutes the initial presentation [7,8]. Differentiating secondary cutaneous involvement by systemic FL from primary cutaneous follicle center lymphoma is clinically important because these entities differ in staging, prognosis, and management [7,8].

Cutaneous manifestations of FL can closely resemble benign inflammatory or autoimmune skin disease, particularly when they present as chronic subcutaneous nodules, plaques, or panniculitis-like lesions [7,9]. Diagnosis may therefore be delayed, especially in the absence of substantial nodal involvement or characteristic systemic symptoms [1,2]. Histopathological examination with immunohistochemistry is essential in such cases to establish the diagnosis and exclude alternative entities, including primary cutaneous B-cell lymphomas and subcutaneous panniculitis-like T-cell lymphoma [2,7,9].

We report a case of systemic FL in which chronic panniculitis-like cutaneous lesions delayed the diagnosis until histopathological and immunohistochemical evaluation identified the underlying malignancy. The case illustrates the need to consider lymphoma in patients with persistent or treatment-refractory subcutaneous lesions [2,7,9].

## 2. Case Presentation

A 52-year-old man with a history of type 2 diabetes mellitus presented with marked unintentional weight loss, iron-deficiency anemia, and elevated inflammatory markers. He also had panniculitis-like lesions of the scalp. Abdominal computed tomography (CT) showed an approximately 100-mm bundle-like infiltration of the mesenteric adipose tissue, raising suspicion of mesenteric panniculitis. Initial gastroenterological evaluation found no underlying malignancy. Because systemic inflammation and the radiological findings persisted, he was referred for immunological evaluation, and IgG4-related mesenteric panniculitis was initially suspected. The patient entered our investigation on 21 February 2025.

Initial treatment with colchicine and methylprednisolone produced partial clinical improvement. Over the following months, however, the cutaneous lesions recurred and progressively worsened. Azathioprine was added, followed by mycophenolate mofetil after the back lesions progressed. Two off-label rituximab infusions produced no sustained clinical improvement. Skin biopsy showed lymphocytic infiltration with lobular features consistent with panniculitis but no evidence of malignancy, and cyclosporine was started empirically. Despite these treatments, the cutaneous lesions remained refractory, prompting further evaluation for an underlying neoplastic process.

**Figure 1:**
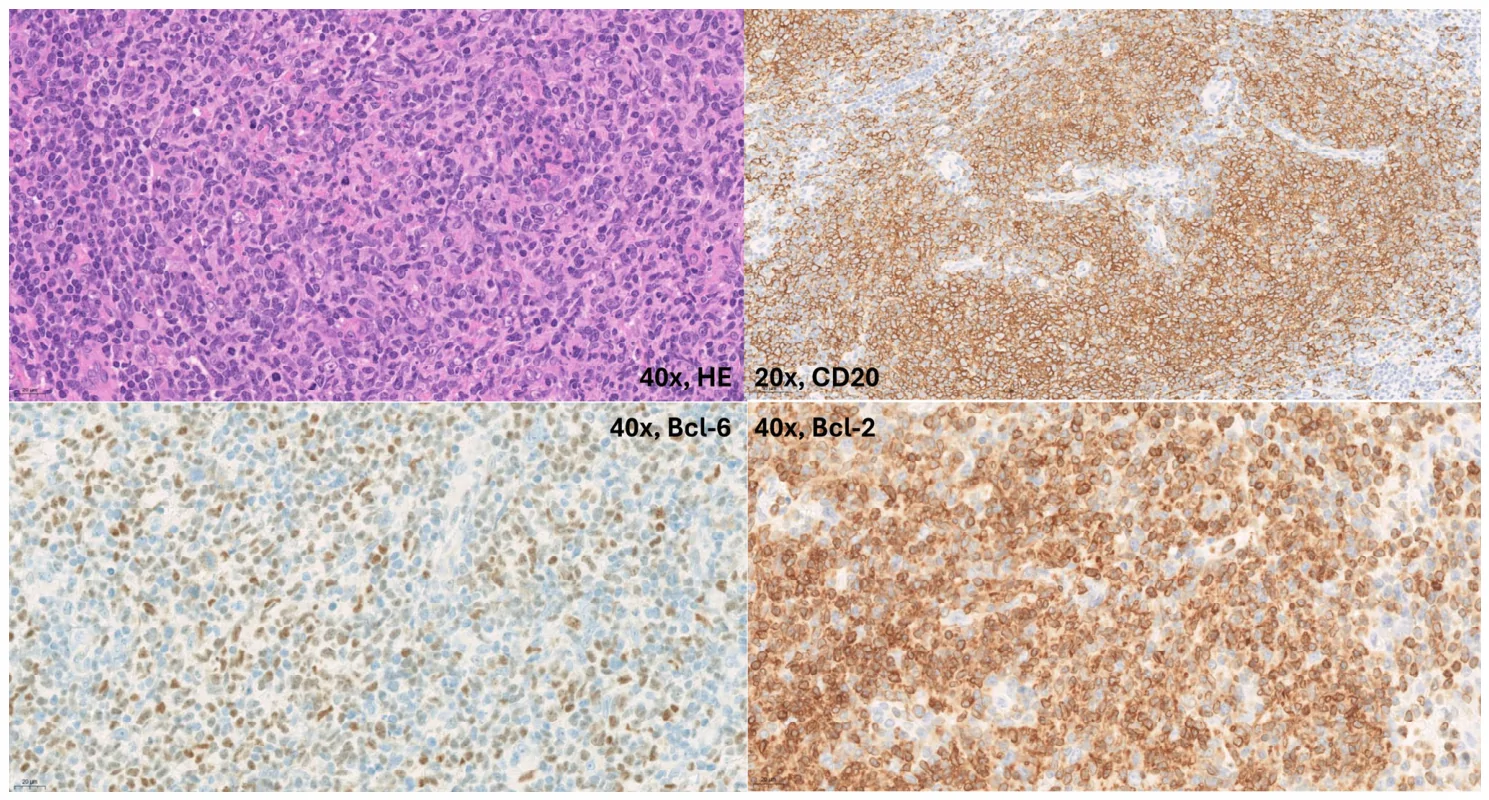
Histopathological and immunohistochemical findings in the excised cervical lymph node. Hematoxylin and eosin staining (×40) and immunohistochemistry demonstrate expression of CD20 (×20), BCL6 (×40), and BCL2 (×40) by the neoplastic B cells.

Following treatment failure, the patient was re-evaluated for occult malignancy. In January 2025, imaging revealed cervical lymphadenopathy, and ultrasonography showed abnormal nodal architecture. Core needle biopsy was nondiagnostic; therefore, an excisional lymph node biopsy was performed. Histopathological examination showed a nodular lymphoproliferative infiltrate composed of heterogeneous medium-to-large lymphoid cells with predominantly centroblastic morphology. On immunohistochemistry, the neoplastic B cells were positive for CD20, BCL2, and BCL6 and negative for cyclin D1 and SOX11. The Ki-67 proliferation index was approximately 15–20% within the nodules. These findings established the diagnosis of grade 3A FL (Figure 1).

Staging with fluorine-18 fluorodeoxyglucose positron emission tomography/computed tomography (18F-FDG PET/CT) demonstrated FDG-avid cervical and supraclavicular lymphadenopathy, involvement of the nasal cavity and nasopharynx, and multiple hypermetabolic cutaneous lesions. Noncontiguous extranodal involvement of the skin, nasal cavity, and nasopharynx established stage IV disease; marked weight loss conferred the B designation. The Groupe d’Etude des Lymphomes Folliculaires (GELF) criteria for treatment were met because of marked weight loss, anemia, and a bulky extranodal lesion measuring approximately 100 mm [6].

**Figure 2:**
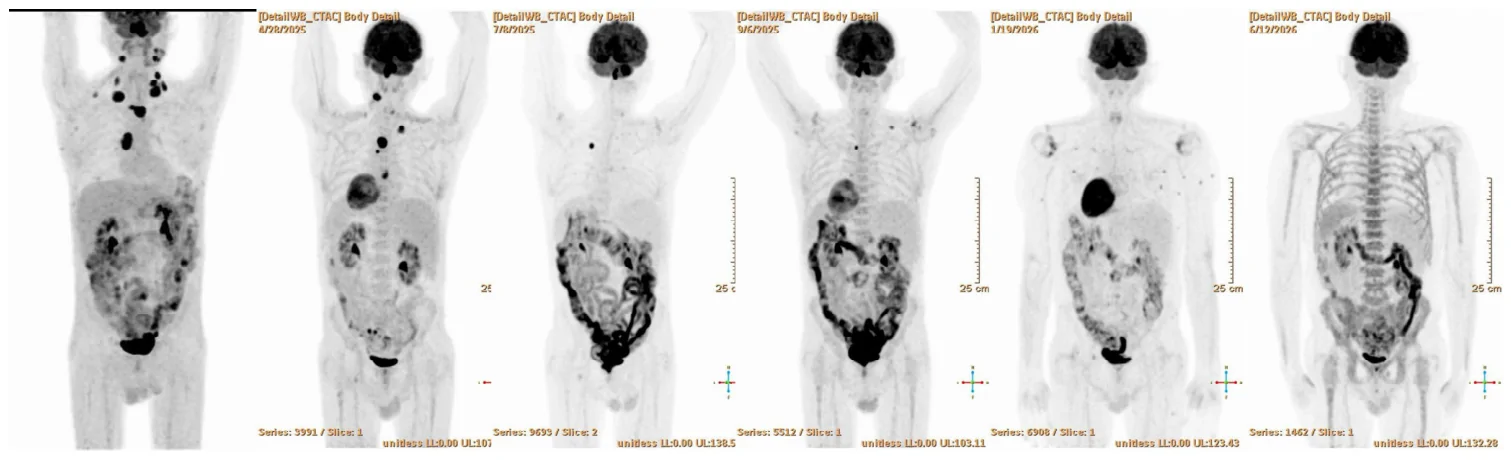
Serial fluorine-18 fluorodeoxyglucose positron emission tomography/computed tomography (18F-FDG PET/CT) images showing the metabolic response from initial staging through day +100 after autologous stem cell transplantation.

R-CHOP chemotherapy (rituximab, cyclophosphamide, doxorubicin, vincristine, and prednisolone) was initiated in February 2025 and administered for six cycles through June 2025; red blood cell transfusions were required. Interim PET/CT after four cycles showed a partial metabolic response, with complete or partial resolution at most evaluable sites. Progressive metabolic activity persisted in the back skin, left submandibular region, and nasal cavity. End-of-treatment PET/CT in July 2025 showed a partial metabolic response (Deauville score 5), with persistent intensely FDG-avid disease in the nasal cavity and nasopharynx (Figure 2).

Persistent FDG-avid extranodal disease after first-line therapy prompted inpatient admission in July 2025 for further evaluation, biopsy, and hematologic support. Biopsy of the nasopharyngeal lesion confirmed persistent FL without a large B-cell component. Salvage chemotherapy was initiated with R-DHAP (rituximab, dexamethasone, high-dose cytarabine, and cisplatin) and continued with R-DHAOx (rituximab, dexamethasone, high-dose cytarabine, and oxaliplatin). Although no renal toxicity occurred, cisplatin was replaced with oxaliplatin at the treating physician’s discretion. After the second salvage cycle, stem cell mobilization and collection yielded 7.4 × 10^6^ CD34-positive cells/kg.

Restaging after salvage chemotherapy showed residual disease in the nasal cavity and right supraclavicular region. Repeat biopsy of the nasal lesion, performed to exclude transformation or infection, confirmed persistent grade 1–2 FL without evidence of transformation. Radiotherapy was then delivered to the nasal cavity/pharyngeal region and right supraclavicular nodal region to a total dose of 36 Gy in 18 daily 2-Gy fractions. During pretransplant evaluation, cholelithiasis with inflammatory changes necessitated elective cholecystectomy before ASCT. Laparoscopic cholecystectomy in December 2025 was converted to an open subtotal procedure because of a contracted gallbladder and dense hilar fibrosis. A postoperative bile leak was treated by endoscopic retrograde cholangiopancreatography and biliary stenting. Histological examination showed chronic fibrosing cholecystitis with acute ulcerophlegmonous exacerbation and no malignancy. Pretransplant PET/CT showed a complete metabolic response. Given this response, the transplant team considered the disease sufficiently treatment-sensitive for consolidative ASCT and recommended proceeding after multidisciplinary assessment of the risks and benefits.

The patient underwent ASCT in March 2026. Conditioning with R-BEAM (rituximab, carmustine, etoposide, cytarabine, and melphalan) was followed by infusion of autologous peripheral blood stem cells on 4 March 2026. Early complications included gastrointestinal mucositis and a single febrile episode; blood cultures remained negative, and empirical broad-spectrum antibiotics were administered. Neutrophil and platelet recovery occurred on days +9 and +10, respectively. At day +100, PET/CT confirmed complete metabolic remission, with no FDG-avid lymphoma.

**Figure 3:**
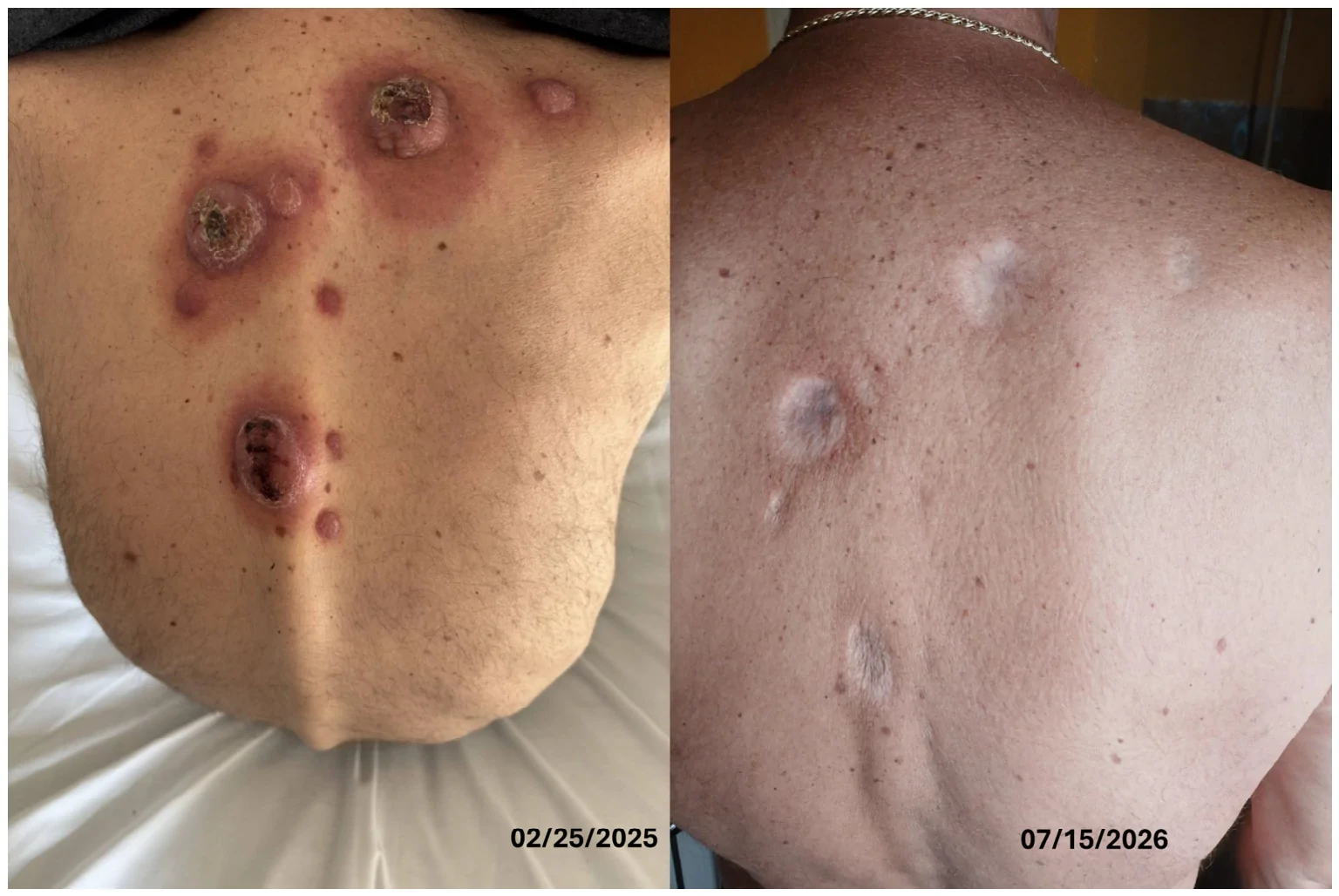
Clinical appearance of the cutaneous lesions during active disease (**left**, 25 February 2025) and after achievement of complete metabolic remission (**right**, 15 July 2026).

At the most recent follow-up in July 2026, the patient remained clinically stable in complete metabolic remission, and the cutaneous lesions had resolved (Figure 3). Hematologic recovery was sustained, antimicrobial prophylaxis had been discontinued, and surveillance follow-up was scheduled for October 2026.

The manuscript was drafted with the assistance of Jenny.AI, but the intellectual content and research were solely the work of the authors.

## 3. Discussion

This case describes systemic FL with an atypical inflammatory presentation. Mesenteric infiltration suggestive of panniculitis and persistent cutaneous and subcutaneous lesions preceded the diagnosis of lymphoma [10]. Although FL usually presents with nodal disease, extranodal or cutaneous involvement may mask the underlying malignancy when it resembles an inflammatory or autoimmune disorder [1,2,6]. In our patient, the lesions responded poorly to sequential immunosuppression, and subsequent histological examination confirmed FL. This sequence suggests that the inflammatory manifestations were associated with the lymphoma, although a direct paraneoplastic mechanism cannot be established [10].

The initial skin biopsy showed lobular lymphocytic inflammation without malignancy, but the lesions progressed despite immunosuppressive treatment. Lymphomatous processes may resemble benign panniculitis clinically and histologically, and transient improvement with corticosteroids does not exclude malignancy [9]. Persistent or recurrent lesions should prompt diagnostic reassessment and repeat tissue sampling [9,10]. When FL is suspected, adequate tissue is also needed to establish the histological grade and exclude large-cell transformation [2,6].

The differential diagnosis included secondary cutaneous involvement by systemic FL and primary cutaneous B-cell lymphoma [7,8]. Subcutaneous panniculitis-like T-cell lymphoma, lupus panniculitis, and IgG4-related disease were also considered because each may resemble inflammatory panniculitis clinically or histologically [9,10]. In this patient, cervical and supraclavicular lymphadenopathy, nasal and nasopharyngeal involvement, and FDG-avid skin lesions favored systemic FL. Correlation of lesion distribution, systemic imaging, histopathology, and immunophenotype was essential to avoid misclassification [2,6,7].

Primary cutaneous follicle center lymphoma is defined by skin-limited disease at presentation, and its distinction from systemic FL with secondary cutaneous involvement rests on staging together with immunophenotypic and molecular findings [5,7]. The panniculitis-like presentation also raised the possibility of subcutaneous panniculitis-like T-cell lymphoma, a distinct cutaneous T-cell lymphoma characterized by subcutaneous involvement without extracutaneous disease at diagnosis [7,9]. Its exclusion is supported in our patient by the B-cell phenotype and by the concurrent nodal, nasal, and nasopharyngeal involvement.

The treatment course further emphasizes the importance of response-adapted reassessment in advanced FL. Although first-line R-CHOP produced an overall partial metabolic response, persistent FDG-avid extranodal disease in the nasopharyngeal region and cutaneous sites raised concern for refractory disease and required histological confirmation [2,6]. Repeat biopsy was performed to exclude large B-cell transformation or infection before salvage therapy [2,6]. In this patient, persistent FL infiltration was confirmed without evidence of a large-cell component, supporting the decision to pursue platinum- and cytarabine-based salvage chemotherapy, local irradiation of residual disease, and consolidation with ASCT [11,12,13].

The role of ASCT in FL has become increasingly individualized in the era of rituximab and new agents [11,13]. However, it remains a relevant consolidation strategy for selected patients with relapsed or refractory disease, particularly when adequate disease control can be achieved prior to transplantation. Pretransplant PET/CT status is prognostic because persistent metabolic activity before ASCT is associated with inferior progression-free survival [12]. In this patient, the response to salvage chemotherapy, followed by a complete metabolic response after local radiotherapy, demonstrated sufficient treatment sensitivity to support consolidation with ASCT [11,12]. CAR T-cell therapy for FL was not available in Hungary at the time of treatment and was therefore not a feasible alternative. PET/CT on day +100 demonstrated complete metabolic remission, indicating short-term disease control. However, longer-term follow-up is needed to assess the durability of the remission.

This case underscores the need for diagnostic caution in cases of persistent, recurrent, or progressive panniculitis-like lesions despite appropriate immunosuppressive therapy [9,10]. In such cases, repeat tissue sampling should be considered, particularly in the presence of systemic symptoms, unexplained elevation of inflammatory markers, lymphadenopathy, or PET/CT-positive lesions [2,6]. Small or superficial biopsies may be insufficient when lymphoma is suspected. Excisional lymph-node biopsy should be considered when an involved node is accessible [2,6]. Early recognition of lymphoma-associated inflammatory manifestations may reduce diagnostic delay and allow timely lymphoma-directed treatment [2,10].

## 4. Conclusions

This case highlights an unusual presentation of systemic FL that initially mimicked mesenteric and cutaneous panniculitis. Persistent or recurrent panniculitis-like lesions, particularly when associated with systemic inflammation, lymphadenopathy, or PET/CT-avid disease, should prompt diagnostic reassessment and repeat tissue sampling. Adequate histopathological and immunohistochemical evaluation remains essential to distinguish inflammatory disease from lymphoma-associated manifestations and to guide timely lymphoma-directed treatment.

## Data Availability

No new data were created or analyzed in this study. Data sharing is not applicable to this article.
